# The Foreign Journals

**Published:** 1836-01

**Authors:** 


### Art. XIV.—THE FOREIGN JOURNALS.

As the readers of the British and Foreign Medical Review will be so much indebted to the contemporary journals of foreign countries, one of our departments being almost exclusively supplied from them, it will doubtless be interesting to many to be made acquainted with their general nature and character. It is therefore our intention to give a series of brief notices of all such publications as come into our hands, or are used by us as materials for the construction of the Third Part of this Journal. We shall arrange the Journals according to the countries in which they appear, or the languages in which they are written; and shall continue to notice a few in each number of the Review, until we have embraced the whole. This will be found, we suspect, not so easy a task as many of our readers, who have not paid attention to the fertility of the medical press in other countries, may be inclined to believe.

### THE GERMAN JOURNALS.

Germany has always been famous for the activity of its press, in every department of literature; and in the number and variety of its periodical publications, of a scientific and literary kind, it takes precedence of all other countries. This statement is equally true in regard to our own profession, the number of books, pamphlets, and journals of all kinds that appear on medicine being immense. The extraordinary fertility of the German press, however, is not owing entirely to the superior desire of the German people for literary gratification, although this is at least equal to that of any other nation, but partly also to the vast extent of the countries in which the German language is spoken, their subdivision into so many independent states, and the consequent existence of so many rival universities and medical schools.

In speaking of the German medical journals in the mass, we must, in justice, bestow our approbation upon them. Almost all of them are edited by men of great learning, many of them by some of the most distinguished physicians and surgeons in Germany; and although, where the channels of communication are so numerous, there must often be a dearth of matter of real value or interest, still it is surprising how very much of sterling science and practical knowledge is to be found in almost every number of every journal. It is true that many of the journals are on a smaller scale than ours; and that the great learning of the editors enables them to make great use of the productions of their contemporaries in foreign countries: still the amount of original communications is very striking, and that they are often of no slight value and interest we trust the Third department of the successive numbers of this Review will evince. In making our selection for this department, we shall unquestionably have to set aside much that was hardly worth publishing, some things that are positively bad, and a good deal that would be unsuited to the taste of English readers; but, in making this statement, we are aware that it is equally applicable to the periodical literature of every country, and to none more so than our own. In conclusion, we are bound to confess, that until we were led by our duties as Journalists to examine, more critically and much more extensively than we had done before, the medical periodical literature of Germany, we entertained a disproportionate estimate of its worth. By way of amends, it will henceforth be no less our pleasure than it will be our interest, to endeavour to diffuse among our readers and the medical profession generally in these countries, a more thorough and extensive knowledge of the vast treasures possessed by our respected brethren in all the German states, than it was possible to obtain from any British publication before the establishment of this Review.

The actual number of Medical Journals now published in Germany cannot be stated with perfect accuracy, as new ones make their appearance, and others become extinct, in every quarter of a year. In Leopold Voss's Bibliotheca Physico-Medica, published in January last, the *Journalistik,* or bill of Scientific and Medical Journals for 1835, amounts to fifty-two, of which number thirty-seven are devoted to the various branches of medical science.

In noticing the Journals, we shall follow no particular order or arrangement, but take them as they first come to hand.

1.—*Journal der practischen Heilkunde.* Herausgegeben von C. W. Hufeland, und E. Osann. *Berlin.*

*Journal of Practical Medicine.* Edited, &c.

This is one of the oldest medical journals in Germany, and the name of one of its editors, at least, has been familiar to the medical profession in all countries, during upwards of half a century. The journal is of small extent, being in small 8vo., and containing only about eight sheets, or 128 pages in each number. It is printed in the Roman character, is published monthly, six numbers constituting a volume. The number now before us is for September, 1835, being the third number of vol. LXXXI. Like most of the German journals, it is not sold in single numbers, but by the year; and it costs, for this period, five rix-dollars and sixteen groschen (between fifteen and sixteen shillings *). It consists entirely of original communications, and has been the medium of conveying to the profession in Germany and other countries, during a long series of years, much valuable information. Hufeland and Osann's journal tells of its antiquity in the humble style of its paper and cover, and general mode of *getting up,* to use the bookseller's phrase.

2.—*Die Bibliothek der Praktischen Heilkunde.* Herausgegeben von C. W. Hufeland, und E. Osann. *Berlin.*

*The Library of Practical Medicine.* Edited, &c.

This little work, although sold separately, is, properly speaking, a part of the journal last noticed. It is in fact the *Review department* of the Journal of *Practical Medicine,* and contains no original communications. It is just half the size and half the price of its fellow, and is published at the same periods. It is printed in the Roman character.

3.—*Journal der Chirurgie und Augen-Heilkunde.* Herausgegeben von C. F. v. Gräfe, und Ph. v. Walther. *Berlin.* 8vo.

*Journal of Surgery and Ophthalomology.* Edited, &c.

This is also an old journal, the number before us being the second of vol. XXIII. It is needless to observe, that its editors are two of the most eminent men in Germany. Although noticing more particularly

* The value of the German *thaler* (rix-dollar, or dollar,) is somewhat less than three shillings English, our pound sterling being worth 6½ Prussian *thalers.*

subjects of surgery and diseases of the eye, as its title imports, this journal by no means restricts itself to such, but often contains valuable papers on the other branches of the science, and many in practical medicine. Like many of the German journals, it frequently gives plates illustrative of subjects contained in it, but we must say that these are, in general, in an inferior style of art. The paper, and general appearance of the journal, belong also to the old school of journalism. It scarcely gives what are properly termed reviews of books, but it gives brief notices of many foreign works, and devotes a few pages to characterize, in a sentence or two, such books as are presented to the editors. It consists of from eleven to twelve sheets, or 180 or 190 pages; it is not published at fixed times, but four numbers at least appear annually, constituting a volume, which is sold for four dollars, or twelve shillings, nearly. It is in the Roman type.

4.—*Neue wissentschaftliche Annalen der gesammten Heilkunde.* Herausgegeben von Dr. J. F. C. Hecker. *Berlin.* 8vo. *Monthly.*
*New Annals of Medical Science.* Edited, &c.

This is a long established journal, having reached its thirteenth volume at the close of last year, when a second series commenced, indicated by prefixing the word *New* to the old title. Its editor is Professor of Medicine in the Berlin university, and is the well-known author of the histories of the Epidemics, so well translated by Dr. Babington. With the present series, a new numeration of the volumes began, and the number now before us is the second of the second volume, but for what month we cannot precisely say, as this journal, like many others of the German journals, contains on its title-page no nearer indication of its publication than the date of the year. The Annals of Professor Hecker is an excellent journal, and in every respect, mechanically as well as in a literary point of view, is executed with great taste and neatness. It is small, each number containing only about eight sheets, or 128 pages; four numbers constitute a volume, and cost two dollars sixteen groschen. It is arranged very distinctly, much on the same plan as the old Medical and Physical Journal of London, with a first department of original papers, and a second of critical notices. In general, the original communications do not exceed three or four in each number, but they are evidently well selected, and often of great value. The critical department is much more extensive, and takes in a wide range of subjects and languages. In the numbers before us we perceive reviews of recent works in Polish, Italian, French, and English, besides German; and we observe that the editor pays particular attention to English works, as we find in these numbers pretty full reviews of the recent works of Hope, Lee, Wardrop, Carswell, &c., the authors of which articles exhibit a perfect acquaintance with the language in which the books are written, as well as with the subjects of them. We shall not be able to transplant into our pages a great variety of matter from the pages of Dr. Hecker's Annals, but what we find there we confidently expect to be good.

5.—*Medicinische Jahrbücher des kaiserl. königl. Österreichischen Staates.* Herausgegeben von Dr. A. J. Freyherrn von Stifft, und redigirt von Dr. Joh. Nep. Edlen von Raimann. 8vo. *Wien.*
*Medical Annals of the Austrian Empire. Vienna.* Edited, &c.

This journal is edited by Drs. Stifft and Raimann, physicians to the emperor of Austria, and they number among their *collaborators* many or most of the medical, surgical, and veterinary professors of the university of Vienna. Each number contains about ten sheets, or 160 pages. It is neatly printed in the Roman type, on middling paper, and is altogether pretty well got up. It appears monthly; six numbers complete a volume, which cost four dollars eight groschen.

These Annals bear somewhat of an official stamp, and contain:—1. Official regulations concerning medical police, and the study of medicine. 2. Accounts of the public health, and of the statistics of the Austrian empire, including minute details respecting the performance of vaccination. 3. Accounts of the prevailing type of disease. 4. Observations in natural history. 5. Medical essays and cases. 6. Reviews of books.

Some of the official rescripts are curious enough: thus, in pursuance of the decree of the court of Chancery of the 31st October, 1833, the use of plum kernels is forbidden in the manufacture of imitation coffee, as being unwholesome; and in another place, the importation of Dr. Struve's artificial mineral waters is prohibited, without any reason being assigned.

Among the peculiarities of this journal we may reckon the large space devoted to statistics, and medical police and jurisprudence; as well as the fact of its being limited to Austrian medicine; this limitation extends, though not without a few exceptions, even to the notices of books. Hence the reviewers, being cramped in their choice of subjects, take account of inaugural theses, &c. &c.

The *Medical Annals of the Imperial and Royal Austrian State* will furnish us with many valuable articles for our third department.

6.—*Allgemeines Repertorium der gesammten deutschen Medizinisch-chirurgischen Journalistik.* Herausgegeben von C. F. Kleinert, M.D., Ausserordentlichem Professor der Medizin an der Universität zu Leipzig, &c. 8vo. *Leipzig. Monthly.*

*General Repertory of the whole Medico-chirurgical Journalistics of Germany.* Edited, &c.

We are obliged to coin a word, to give even a bad translation of the title of this singular and truly German journal, with a brief account of which we shall, for the present, conclude our notices of the periodical medical literature of Germany. This is a large journal, and professes nothing less than to give an epitome of the whole original matter of all the German medical journals, which amount to at least forty; exclusive of a dozen more, devoted to the various sciences. The *Repertorium* has existed nine years. It is published monthly, and the number now before us bears the date of *August;* but, by a postscript, we observe that it was not in reality published until the 9th of October, and as the other numbers bear similar rectifications, we must infer that the industry of even a German editor may find tasks too hard for it. It is published in monthly parts, each part containing about eleven sheets and a half, or 184 pages, and every number has its own numeration of pages. The twelve monthly numbers, constituting the *Jahrgang*, cost seven dollars.

The plan of this journal is really excellent, and it must be extremely convenient to those who wish to know what is going on in the wide field of German medical literature, and find it impossible to refer to all the original publications. We would therefore recommend it to such of our readers as take an interest in German literature, and only desire to take in a single journal. It is precisely on the same plan as the English journal published some years since in London, entitled, *The Medical Intelligencer.* Each journal is taken in turn, and every paper in each journal is epitomized with much fidelity, and, it must be, with great labour and industry. The inferior articles are passed over slightly, but of the best communications the analysis is frequently so full as to convey all that is really of importance in the original.

To give a more precise idea of the way in which this journal is composed, we may state, that of the two last numbers now before us, that for *July* devotes 25 pages to *Rust's Magazin;* 26 to *Clarus und Radius's Beiträge;* 18 to *Pfaff's Mittheilungen;* 6 to *Schmidt's Jahrbücher;* 20 to *Dzondi's Aeskulap;* 12 to *Trousseau's Allgemeines Journal;* 24 to the *Medizinische Zeitung* of Berlin; and 26 to the *Austrian Jahrbücher;* while that for *August* gives 9 pages to *Müller's Archiv;* 10 to the *Annalen der Pharmacie;* 20 to *Clarus und Radius's Beiträge;* 15 to *Pfaff's Mittheilungen;* 30 to *Horn und Wagner's Archiv;* 20 to *Von Ammon's Zeitschrift;* 30 to *Von Gräfe und von Walther's Journal;* 27 to *Gerson und Julius's Magazin;* and 16 to the *Medizinische Zeitung.** We shall sometimes avail ourselves of the compendious information supplied by the Repertorium, more particularly in regard to such journals as do not come at all into our possession, or reach us irregularly, or too late.

### ITALIAN JOURNALS.

Italy, like Germany, possesses a *Journalistik* very large in relation to its population, and partly owing to the same causes, viz. its subdivision into various states, and the great number of its rival universities and schools of medicine. A good deal also depends on the activity and zeal with which medicine is cultivated in Italy. Including those of Sicily, the number of journals now published in the Italian language may amount to about fifteen or twenty. We shall, at present, only notice two or three of these; and indeed we have to lament the great difficulty with which they are obtained in England. At least we are still *expecting* several which were ordered more than six months since; and we find that some of our friends, readers of Italian, have been equally unfortunate in their commercial speculations. Owing to the existence of so many different states in Italy with different laws and fiscal regulations, the conveyance of packets and letters from one part of the peninsula to another is extremely uncertain, and even the ignorance in one state or city of what is doing in another at no great distance, is very remarkable. It is too true, as our respectable bookseller at Florence, Signor Molini, writes to us, that "a Roma non si sa cosa si fa in Napoli; a Milano s'ignora quel che si fa in Roma."

In speaking of such of the Italian journals as we have had an opportunity of examining, we are bound to confess, that, taken as

* Besides the analysis of the journals, each number of the Repertorium contains a classified catalogue of all the books reviewed in the various journals, with occasional brief notifications of the opinions of the reviewers.

a whole, they are inferior in value both to the French and German journals. Although we have too much reason to criticise the occasional verbosity, fanciful hypotheses, and inconsequential reasonings to be met with in those last named, we are sorry to say that the Italians are, if any thing, still more verbose, more fancifully hypothetical, and more inconsequential. Numerous exceptions, however, are to be met with, and we doubt not but we shall from time to time have to present to our readers, among our selections from the Italian journals, papers which would do honour to the profession in any country.

1.—*Antologia Medica.* Di Val. L. Brera, D.M.C., &c. &c. *Venezia. Monthly.*
*Medical Anthology.* Edited, &c. *Venice.*

Properly speaking, this journal ought not perhaps to be entered in our list, as, although only commencing with 1834, it ceased to be published last summer, in consequence of the illness of the editor, but it is probably once more in existence, as a new series of it has been announced for publication in January, 1836. Our readers need not be told of the eminence of the editor, as his name has been familiar to the profession for many years past; and when we state that the enunciation of his honorary titles literally fills one whole close-printed page of his journal, it will be allowed that his eminence has not been overlooked by his brethren in foreign countries. He was long professor at Padua, but has been within these few years removed to Venice. The Antologia is a sort of continuation of journals formerly published by the editor in other parts of Italy. It is a large journal, published monthly, and consisting partly of original communications, and partly of reviews of books. It has hitherto contained a good many original papers, some of considerable value; and perhaps it may be regarded, on the whole, as one of the best of the Italian journals. Its form is what our booksellers term royal 8vo.; each number contains about six sheets, or 100 pages. Six numbers constitute a volume, and are sold for fourteen Austrian *lire.* The number for January, 1834, contains a fine portrait of the editor. We shall notice the new series of the Antologia on its appearance.

2.—*Annali Universali di Medicina.* Compilati dal Signor Dottore Annibale Omodei. 8vo. *Milano.*
*Universal Annals of Medicine.* Compiled, &c.

This is the best known, and one of the oldest, if not the oldest of the Italian journals.* It must also be regarded as one of the best, although it frequently contains much more matter in appearance than in reality. Like many Italian books, the margin is vastly disproportioned to the printed *form,* the paper being royal 8vo., the book only small 8vo. It is published monthly at Milan, each number consisting of about twelve sheets, or 192 pages. Three numbers form a volume, and the four annual volumes are sold at the place of publication for thirty-six Austrian *lire.* It consists of original memoirs, reviews, and selections from other journals, foreign and domestic. English literature is not overlooked: for instance, the number for August, 1835, now before us, contains one of Dr. Stokes's excellent lectures on nervous diseases, delivered in

* The number now on our table is 225, being the last of vol. LXXV.

Dublin, in the winter of 1833-4, and published in the Medical and Surgical Journal, also a pretty full review of the first part of vol. xviii. of the Medico-Chirurgical Transactions of London. We are sorry to say, that the recent numbers which we have seen of the journal do not contain much original matter of importance. We have, however, derived something from them for our department of selections, and expect to continue so to do.

3.—*Giornale delle Scienze Medico-Chirurgiche.* 8vo. *Pavia.*
*Journal of the Medico-Chirurgical Sciences.*

This journal only commenced in July, 1834, and we have received the first nine numbers of it. It is published monthly at Pavia, and is of small size, containing only three sheets, or forty-eight pages, and costing, at the place of publication, only seven Austrian *lire* per annum. It is on the more common plan of medical journals, composed partly of original communications, partly of reviews of books, and partly of selections from other journals, foreign and domestic. Hitherto this journal has not presented in its pages much original matter of importance. The name of the editor is not given.

### FRENCH JOURNALS.

Although the whole of the journals published in the German language exceed very considerably in number those published in French, or any other language, still we believe the medical journals of France are much more numerous than those of any other single nation. Compared with those of our own country, the number and variety of the medical journals published in France must appear very striking; and we here call attention to the fact, as an important indication or illustration of the difference of national character, as well as of the difference in the habits, tastes, and feelings of the members of the medical profession in these two neighbouring nations. In giving the catalogue of the French journals, we regret that it is not in our power to state the number of copies of each published, as it is only by knowing this that any just conception could be formed of the relative number of medical readers in the two countries. We think, however, it will not be doubted for a moment by those who peruse the two following lists of the journals published in the United Kingdom and Paris alone, that the numbers at least are greatly on the side of our neighbours. As we cannot adduce the number of copies published of the French journals, we shall refrain from stating what is, or is supposed to be, the circulation of the British journals.

### BRITISH JOURNALS.

| | Price per Annum. s. | d. |
|---|---|---|
| *Quarterly.* | | |
| 1. The Edinburgh Medical and Surgical Journal | 24 | 0 |
| 2. The Medico-Chirurgical Review | 24 | 0 |
| *Once in two Months.* | | |
| 3. The Dublin Medical and Chemical Journal | 21 | 0 |
| *Weekly.* | | |
| 4. The Lancet | 34 | 8 |
| 5. The Medical Gazette | 34 | 8 |
| 6. The London Medical and Surgical Journal | 26 | 0 |

### FRENCH JOURNALS.

| | | Francs per Annum. |
|---|---|---|
| | *Annual.* | |
| 1. | Répertoire Annuel de Clinique Médico-Chirurgicale | 8 |
| | *Quarterly.* | |
| 2. | Mémoires de l'Académie Royale de Médecine | 20 |
| 3. | Annales d'Hygiène et de Médecine Légale | 26 |
| | *Monthly.* | |
| 4. | Archives générales de Médecine | 26 |
| 5. | Archives de la Médecine Homœopathique | 18 |
| 6. | Journal et Bulletin de Pharmacie | 15 |
| 7. | Journal de Chimie Médicale, de Pharmacie et de Toxocologie | 14 |
| 8. | Revue Médicale Française et Etrangère. *Paris.* | 27 |
| 9. | Bulletin Bibliographique des Sciences Médicales | 3 |
| 10. | Journal des Connoissances Médicales | 6 |
| 11. | Journal des Connoissances Médico-Chirurgicales | 10 |
| 12. | Journal de Médecine et de Chirurgie pratiques | 10 |
| 13. | Journal de Pharmacie et des Sciences accessoires | 15 |
| | *Once a Fortnight.* | |
| 14. | Bulletin de Thérapeutique Médicale et Chirurgicale | 15 |
| | *Weekly.* | |
| 14. | Journal Hebdomadaire des Progrès des Sciences Médicales | 20 |
| 15. | Gazette Médicale de Paris | 40 |
| 16. | Journal de Santé, à l'usage des gens du monde | 10 |
| | *Three times a Week.* | |
| 17. | La Lancette Française, Gazette des Hôpitaux | 36 |

Considering the Parisian journals generally, we feel bound to say that they exhibit a great deal of talent, and a still greater degree of zeal in the writers and editors. Many of them contain a regular succession of original memoirs of extreme value, both in a scientific and practical point of view, and all of them present us, from time to time, with papers of much interest. Not a few of them, it must be allowed, publish much which can be interesting to few but the writers; and a still greater number exhibit frequent specimens of speculations or surmises announced as facts, of visionary hypotheses recorded as theories, and of inferences and conclusions most lame and impotent: still it is true, as already stated, that, taken as a whole, the periodical medical press of Paris is not only conducted with great talent, but fraught with an infinite deal of practical knowledge, and powerfully promotive of the progress of every branch of medical science. The department in which it is most defective, and in this respect the whole continental periodical press is in the same predicament, is that of *reviewing.* In this particular, the British journals are greatly in advance, and, indeed, the continental nations do not seem fully to understand this branch of literature, as it is practised in England. At least we have never met with a work, in any of the continental languages, whether in the class of medicine or otherwise, which in point of fulness or variety could be justly put in competition with British works of the kind now referred to: we think this a serious deficiency, and doubt not it will be remedied ere long. Some of the German journals come nearer the British standard of Reviews, and we may name, as of this class, the very excellent journal of our learned correspondent Dr. Hecker, of Berlin, the "Neue wissentschaftliche Annalen der Gesammten Heilkunde."

Before closing these few preliminary remarks on the French journals, we must notice a publication which, whatever may be thought of the principles concerned in its establishment, will probably tend more than any thing else to introduce them to foreign countries; we refer to the *Encyclographie des Sciences Médicales,* now pretty well known in this country. This is nothing less than a complete republication of all the French medical journals in a single volume, issuing in monthly volumes from the Brussels press, and sold at the cost of five francs and a quarter each, or about three guineas per annum in this country. The work is printed in the largest royal 8vo. form, with a small type, in double columns, and averages about from eighteen to twenty sheets, or from 280 to 320 pages each volume. The Encyclographie is edited by Dr. Marinus of Brussels, the editor also of a Belgian Journal (Bulletin Médical Belge) circulated under the same cover as the Encyclographie.

At present we shall only notice two or three of the French journals which happen to lie on our table, in their original form, not in the uniform livery of the Encyclographie.

1.—*Revue Médicale, Française, et étrangère, et Journal de Chirurgie, &c.* Par une Réunion de Professeurs, &c.

This journal is published monthly, three numbers forming a volume. It has been established fifteen years, and is a work of much value. It is composed of four or five departments, viz. original memoirs, analyses of books, bibliographical notices of books, notices of the foreign journals, and varieties, domestic and foreign. Many of the original memoirs are excellent, and although the reviews of books are in general too brief, and not sufficiently critical, still the journal, in accordance with its title, certainly gives a more complete account of published works than most of the other journals; and it would be doing injustice to the eminent writers employed in it (and whose names, as is usual on the continent, are subscribed to their articles,) to deny that many of their analytical memoirs are executed in a very excellent manner, and with a praiseworthy spirit of impartiality.

2.—*Archives de Médecine, Journal complémentaire des Sciences Médicales.* Publié par une Société de Médecins.

This is also a well established journal, and one of much merit. It commenced in 1823, and is published monthly, each number containing about 130 or 140 pages, and four numbers constituting a volume. It is, on the whole, arranged on the same general plan as the *Revue,* but contains more original matter, and a less extensive bibliographical department. Its original memoirs are often of great value. Like the *Revue,* and many of its contemporaries in France, as well as in Germany and Italy, the Archives often gives lithographic plates to accompany the memoirs contained in it; but we are compelled, by a regard for truth, to say that these are, in general, not at all proportioned in point of merit to the letter-press they are intended to illustrate, and are, in fact, quite unworthy of the present state of the arts in the French capital.

3.—*Gazette Médicale de Paris.*

This is a comparatively recent publication. Two other journals, the *Gazette de Santé,* and *Clinique des Hôpitaux,* have been incorporated with the Gazette, which appears weekly in the form of a large 4to. sheet of double columns, and without any wrapper. It is edited by M. Jules Guérin. It is a well conducted and excellent journal; nearly on the plan of our own weekly journals, only that it does not publish the lectures, ex cathedrâ, of the professors. To make up for this, it gives very full and sometimes very interesting reports of the memoirs presented to the *Académie des Sciences*, and the *Académie de Médecine*, and of the discussions thence arising among the members of these learned bodies. The Gazette always contains original memoirs, and its *leading article*, as it is called, is generally one of value. It gives a few brief notices of the books published in France, and is remarkable among the French journals by the copiousness of its selections from the foreign journals, particularly those of Germany and Italy. It is from this source that much of the information concerning the medical literature of those nations is conveyed, at second-hand, to this and other countries. As might be looked for from a journal making its appearance after such brief intervals, the *Gazette* properly enters somewhat into the medical politics and professional gossip of the day, but with much restriction and good taste; and indeed it is but a just compliment to the medical periodical press of Paris, to include the whole in this category. In this respect, we are afraid our own periodical literature, general and medical, will derive no credit from a comparison with that of our neighbours. They certainly exceed us in the warmth of their scientific controversies; but we are more personally quarrelsome and bellicose than they are; and in this, we humbly conceive, we derogate from the dignity of the noblest of the sciences.

4.—*La Lancette Francaise, Gazette des Hôpitaux.*

This journal, like the *Gazette*, is in quarto, but instead of appearing weekly in whole sheets, or one sheet and half, it comes out in half-sheets three times a week. Its price is 36 francs a year. In accordance with its title, borrowed, no doubt, from the English journal of that name, the *Lancette* takes much more account of the proceedings at the hospitals than the other journals. It gives numerous cases, contributed by the resident pupils, and likewise frequent abstracts of lectures, for the most part clinical, but sometimes general; but it never supplies such complete courses of lectures as its namesake, and the other weekly journals of London. It gives regular reports of almost all the hospitals in Paris, and particularly of their clinical departments, besides containing some original memoirs by the seniors of the profession. These are, however, comparatively rare; and although this journal is well conducted, and contains much matter of temporary interest, still as it is more calculated to supply the materials for good memoirs than good memoirs themselves, we shall probably draw less from its pages than from some other works of much less interest and indicating less extent of knowledge on the part of their conductors.

5.—*Annales d'Hygiène et de Médecine Légale.*

This journal has existed seven years, and is one of the most valuable published in any country. It is supported and conducted by some of the most able physicians and physiologists of Paris, the names of fifteen of whom are publicly announced. It is published quarterly in octavo, and costs eighteen francs per annum. It seems carefully restricted within the limits contemplated in its title, and contains almost always more than one memoir of decided value and interest. This will be understood when we state that the few last numbers now before us contain communications from Esquirol, Marc, Devergie, Chevallier, Barruel, Orfila, Parent-du-Chatelet, Lombard, &c., and from scarcely any one else. The departments of Hygiene, Medical Jurisprudence, and Medical Statistics, being, in our opinion, of extreme importance, and very inadequately cultivated in this country, we shall draw largely in our *selections* from this journal. We would most willingly recommend to some of our medical brethren the establishment of an English journal on the same plan as the French publication, or rather with the addition of the department of medical statistics; but we are afraid that the taste for medical literature, and medical reading, is not at present sufficiently cultivated or sufficiently general in this country to authorise the risk of such a publication. We trust, however, that the taste is merely in abeyance, not actually wanting; and it has been one great object in the establishment of our own Journal, and will be the constant endeavour of its conductors, to keep in view the extension and improvement of the taste for the better and more substantial parts of medical science.

### DANISH JOURNALS.

We are at present less acquainted with the journals published in the ancient language of Denmark than we could wish, and than we hope to be. It will not be expected, from the small extent of the countries in which the Danish language is spoken, that the medical journals are numerous. We have as yet only received the three of which we shall here give a brief notice: we are unacquainted with their actual number.

1.—*Bibliothek for Læger. Udgivet af Directionen for det Classenske Literaturselskab.* Redigeret af dens medlem C. OTTO, M.D., &c. Small 8vo., *Kjobenhaven.*

*Library for Medical Practitioners.* Edited by Professor OTTO. *Copenhagen.*

Although the journal itself is but little known in this country, the name of its editor is familiar to medical readers in all countries. The journal has been published in its present form since the year 1821. It comes out quarterly, in the form of a small octavo, or large duodecimo, of about 280 pages, and two numbers constitute a volume. It seems a remarkably well planned and well conducted journal. It consists partly of original communications, and partly of extracts from foreign journals, about one half of the number being devoted to each subject. The original communications seem to be valuable, and the extracts carefully selected, and well arranged. We shall certainly profit from the former, by transplanting some of them to our Third department; and we may profit also in the arrangement of this department, by adopting some ot the learned editor's plans. The extracts are made from the German, French, Italian, and English journals. In the numbers before us we observe no reviews or notices of Danish books. In the department of *medical intelligence* at the end of each number is a list of the candidates for licences, who have undergone examinations at the university in medicine, surgery, and pharmacy; and we observe, attached to each name, the candidate's place of residence, and the judgment formed by the examiners of the respective merits of the parties, as shown in their examination. The certainty of this last announcement must, we should think, have some effect on the conduct of the students, previously to going up for examination; as, however pleasant it might be to *Claus Jacob Emil Hornemann*, to see his performance spread throughout the Danish dominions as "laudabilis unanimi consensu," and although even *Hendrik Carl Meinig Oviding* might not be ashamed to have his examination characterized as *laudabilis;* we do suspect that our less fortunate friends, *Christian Drejer*, and *Anton Hermann Gravenshorst*, must with less satisfaction see themselves recorded in the pages of our learned contemporary, with the very dubious compliment of ***haud illaudabilis.***

2.—*Journal for Medicin og Chirurgie. Udgivet og redigeret af Bendz, Haugsted, &c. Kjobenhaven.*
*Medical and Surgical Journal, &c. Copenhagen,* 8vo.

This is a new monthly journal, which commenced January, 1833; we presume that it is still continued, though we have not seen any recent number. It is a small journal, consisting only of six or seven sheets, or about 100 pages. It is published monthly, and four numbers form a volume. It contains original communications, "Hospitals-klinik," reviews of books, selections from foreign journals, and medical intelligence of various kinds. Among other original communications, we observe some by professor Bang; and among the foreign articles we see extracts from the Edinburgh Journal and the Lancet, and from some of the French journals.

3.—***Eyr**, et **Medicinsk Tidsskrift. Christiania.***
***Eyr**, a **Medical Journal.***

Although a Danish journal, *the **Eyr*** is published in Norway, being edited by professor Holst, of Christiania. It has existed since 1826, and has now reached its tenth volume. It is published quarterly, in small octavo numbers, of from 100 to 120 pages, four of which compose the annual volume. It consists principally of hospital reports, and other original communications, which we hope to profit by, more particularly in the statistical section of our selections. We may mention that the word *Eyr* is the name of an ancient Scandinavian deity, having some relationship, at least in function, to the Grecian Hygeia.

### AMERICAN JOURNALS.

The energetic character of the American people, which we feel proud to regard as derived from the same common ancestry as ourselves, and the astonishing progress made by them during the last half century in all the arts and sciences, are no less conspicuous in the actual state of medecine in the United States, than in any of the other branches of human knowledge and social amelioration. Were it not, however, that we are conscious of a firm resolution to make the state of medical science among our North American brethren better known, and more justly appreciated in England, we should almost be ashamed to confess how little we ourselves knew of it, and how little is really known of it by the great majority of even our best informed physicians and surgeons. While the medicine of France is familiar to most men of any education among us, and that of Germany and Italy is known, partially at least, to many of the learned physicians and surgeons of Great Britain and Ireland, particularly the junior members of the profession, the condition of our science throughout the vast territories and in the immense cities of the United States, although recorded in our own language, and cultivated in the same spirit as by ourselves, is scarcely known to us at all. A striking proof of this is afforded by the fact that in some recent histories of medicine published in this country by men of the very first talents and acquirements, scarcely any notice whatever is taken of America, or of the improvements or discoveries for which we are indebted to American physicians and surgeons. An equally striking evidence of the same fact is supplied by the extremely limited importation into this country of books published in America, and by the non-circulation of American journals among us. The converse of the proposition, viz. the extreme eagerness with which English books are received in America, is no less strikingly illustrated by the well-known fact that all good works on British medicine are not only imported into but are immediately republished in America, and circulated in vast numbers. One of the works reviewed in our present number, Dr. Clark's Treatise on Consumption, although not long published in this country, has been already reprinted in America; and another work of which a review was prepared for the present number, but which the pressure of other matter has obliged us to postpone to our next, Dr. Combe's admirable work on Hygiene, has not only been reprinted in America, but circulated to the amount of 10,000 copies. The Medico-Chirurgical Review has been for some years regularly reprinted in America, and enjoys, we believe, an extensive circulation in that country.

The zeal with which medicine is cultivated in America is equally manifested by the number and variety of the medical journals published there; and we are bound in fairness to add, that the original communications and criticisms contained in such of them as we have met with, sufficiently prove that it is not a zeal without knowledge.

Having occupied so much of our space in these general remarks, we shall be able, on the present occasion, to refer particularly to only one or two of the American journals. We may, however, give the titles of several others.

1.—*The American Journal of the Medical Sciences.* Edited by ISAAC HAYS, M.D. *Philadelphia.* 8vo.

This is the American journal best known in England, and is, we believe, one of the best published in America. It is published quarterly, in large handsome numbers, containing eleven or twelve sheets each, and sold in America at five dollars per annum. The periods of publication are February, May, August, and December. Two numbers constitute a volume. The last number that has reached us is XXXII., published in August last, and constituting the second part of the sixteenth volume. The American Journal is divided into four departments, containing original communications, reviews, bibliographical notices, and a quarterly periscope, or selections from foreign or domestic journals. In all these departments, the plan and the execution are alike exemplary. Many of the original communications are valuable, although too many of them consist in the detail of cases to please us. The reviews are in general good, sometimes excellent, and the bibliographical notices, whether of

American or foreign works, seem for the most part impartial, and neatly executed. The periscope, or department of selections, is also well arranged and judiciously filled.

2.—*The North American Archives of Medical and Surgical Science.* Edited by E. GEDDINGS, M.D., Professor of Anatomy and Physiology in the University of Maryland. *Baltimore,* 8vo.

This is a new journal, or rather a new series of a journal, by the same editor. It commenced in October, 1834, and has just completed what the Germans call its first *Jahrgang* with great eclat, and we believe with much success. We are sure that it deserves success. The Archives is not only excellent in plan, but is supplied with much valuable matter, and is certainly one of the neatest and best got up journals we ever saw. Its paper and print are beautiful, and its cover almost too gay for the severity of republican taste. The journal is published monthly; each number consists of four or five sheets; six numbers form a volume, and the annual subscription is five dollars. It comprises the following departments: 1. Original communications. 2. Articles selected, entire or abridged, from foreign journals. 3. Analytical and critical reviews and bibliographical notices. 4. Collectanea, medical intelligence, &c. Our present number contains sufficient proof of the value we set upon this journal, and we have little doubt but we shall often recur, with advantage, to its pages.

The following are the titles of some of the other American journals, of which we hope to give some account in our subsequent numbers.

1. The Boston Medical and Surgical Journal.
2. The United States Medical and Surgical Journal.
3 The Boston Medical Magazine.
4. The Transylvania Journal of Medicine and the Associate Sciences.
5. The Western Medical Gazette.
6. The Western Journal of Medical and Physical Sciences.

In concluding these very imperfect notices of some of our foreign contemporaries, we take this opportunity of stating that we shall be happy to exchange the British and Foreign Medical Review with any Journal, the editors of which may be pleased to transmit it to our London publishers or to their agents, free of expense. The foreign agents named on our cover are authorized to negotiate this exchange for the journals of their respective countries. It is the anxious desire and earnest hope of the Editors of the British and Foreign Review to make it a freer medium of communication, and a closer bond of union, between the members of the medical profession in all civilized countries, than has hitherto existed. It is most delightful to all who cultivate the arts of peace, to live in times when the nations of the earth may freely communicate with one another without restraint or difficulty; and it is doubly delightful to those who, like the members of our profession, are striving only for what is good, to find themselves associated in their labours with the virtuous and the wise of every land, differing indeed in the external and unessential characters of language, customs, and civil polity, but identified in the common desire to improve the physical, moral, and intellectual condition of man, and, consequently, to augment the happiness, and exalt the dignity of the human race.

